# Sex- and Gender-Specific Drug Abuse Dynamics: The Need for Tailored Therapeutic Approaches

**DOI:** 10.3390/jpm13060965

**Published:** 2023-06-08

**Authors:** Susanna Marinelli, Giuseppe Basile, Roberto Manfredini, Simona Zaami

**Affiliations:** 1 School of Law, Università Politecnica delle Marche, 60121 Ancona, Italy; susanna.marinelli@tiscali.it; 2 IRCCS Orthopedic Institute Galeazzi, 20161 Milan, Italy; basiletraumaforense@gmail.com; 3 University Center for Studies on Gender Medicine, Department of Medical Sciences, University of Ferrara, Via Luigi Borsari 46, 44121 Ferrara, Italy; roberto.manfredini@unife.it; 4 Department of Anatomical, Histological, Forensic and Orthopedic Sciences, “Sapienza” University of Rome, 00161 Rome, Italy

**Keywords:** drug use disorders, sex- and gender-specific approaches, neurobiological implications, psychiatric comorbidities

## Abstract

Sex and gender have been gaining ever greater attention due to their associated risks, dynamics, patterns and protective factors underlying substance abuse and addiction. Such differentiations and the clarification of complexities thereof take on even greater relevance in light of drug abuse scope worldwide. According to the 2022 World Drug Report released by the United Nations Office on Drugs and Crime (UNODC), in 2020 an estimated 284 million people worldwide aged 15–64 had used a drug within the last 12 months. The authors have set out to shed a light on determinants and contributing factors of drug abuse based on sex and gender and outline policy and medicolegal remarks aimed at delineating sex- and gender-based approaches towards drug abuse therapeutic interventions that are both therapeutically and ethically/legally viable and grounded in an evidence-based set of standards. Neurobiological data suggest that estrogen may facilitate drug taking by interacting with reward- and stress-related systems. In animal research, the administration of estrogen increases drug taking and facilitates the acquisition, escalation, and reinstatement of cocaine-seeking behavior. From a medicolegal perspective, it is of utmost importance to take into account the whole picture constituting each patient profile, which certainly includes gender factors and contributors, when outlining a therapeutic approach. Failure to do so could lead to negligence-based malpractice allegations, in light of the scientific findings representing best practices with which clinicians need to comply when caring for SUD patients.

## 1. Introduction

According to the 2022 World Drug Report released by the United Nations Office on Drugs and Crime (UNODC), in 2020 an estimated 284 million people worldwide aged 15–64 had used a drug within the last 12 months [1]. This amounts to approximately 1 in every 18 people in that age group, or 5.6 percent, and represents a 26% increase over 2010 levels, when the estimated number of people who used drugs was 226 million and prevalence was 5 percent [1]. This is in part attributable to global population growth. Comparisons over time of such global estimates should take into account their wide uncertainty intervals.

Such numbers reflect rising trends both in use and in substance use disorders (SUDs) over the past decade [1,2]. Substance use is frequently coupled with psychiatric comorbidities; hence, therapeutic and prevention pathways ought to be targeted to both concurrently [2]. Alarmingly, however, prevention and treatment continue to fall short in many parts of the world, with only one in seven people with drug use disorders receiving treatment each year [1].

Sex and gender have been gaining ever greater attention due to the risks, dynamics, patterns and protective factors underlying substance abuse and addiction [3,4]. The so-called “gender gap” in drug abuse and addiction trends has long been observed as showing higher prevalence in male users; such a spread, however, has been shrinking over time [5], and such a development points to the ever-greater relevance of shedding a light on sex and gender differences in substance abuse, particularly from the standpoints of etiology and maintenance. It is therefore worth elaborating on the biological underlying dynamics, epidemiology, and therapeutics of SUDs also by accounting for sex and gender variations. Neurological layout, function and structure, hormonal balance and dynamics, and metabolic mechanisms constitute biological sex differences which have bearing on abuse processes and developments from a biological perspective [4,5]. Adverse medical, psychiatric, and functional consequences associated with SUDs are often more severe in women [3,4,5]. Extremely complex issues may arise relative to substance use as a result of both sex and gender. Sex differences are determined by biological factors, such as sex chromosomes and hormones, whereas gender differences are rooted in culturally characterized roles for men and women, which can also contribute to the onset and progression of substance use and addiction.

## 2. Materials and Methods

The authors have set out to shed light on determinants and contributing factors of drug abuse based on sex and gender and outline policy and medicolegal remarks aimed at delineating sex- and gender-based approaches towards drug abuse therapeutic interventions. A broad-ranging search of scientific sources, including recommendations and guidelines from scientific, regulatory, and law-enforcement bodies and institutions, has been assessed in order to achieve a degree of objectivity and clarity as to how sex-differentiated addiction patterns unfold; a total of 116 sources were ultimately deemed suitable for the purpose of the present article. In light of the article’s fundamental purpose, only sources specifically laying out sex- and/or gender-related differences (not necessarily as the centerpiece of the article) have been included, so as to avoid any “male-bias”, i.e., the tendency of research studies to disproportionately focus on males (whether human or animal studies) that might detract from the thoroughness and comprehensiveness of the findings. Elements contributing to a policy-making and medicolegal analysis necessarily need to rely on sex- and gender- focused analyses in order to inform legislative/regulatory initiatives through evidence-based data and findings.

## 3. Results

Many women with substance use disorders are also diagnosed with other mental disorders, which is important because interactions between mental illnesses can worsen the course and outcomes of both [6,7].

Patients who have both a substance use disorder and another mental health condition often have symptoms that are more persistent, severe, and resistant to treatment compared to patients who have either disorder alone [8].

Both disorders should be treated at the same time, in order to improve the likelihood of success [9]. Substance use disorders may progress differently for women than for men. Women often have a shorter history of using certain substances such as cocaine, opioids, marijuana, or alcohol. Still, by the time women seek SUD treatment, women exhibit more severe medical, behavioral, psychological, and social issues [10]. That seems to point to a shorter time in female SUD patients between first using the substance and the development of addiction/dependence. Although men are more likely than women to report both a mental health and substance use disorder, women are more likely than their male counterparts to suffer from certain mental health conditions, such as depression, anxiety, post-traumatic stress disorder (PTSD), and eating disorders [11].

Research on substance abuse in women ties opioids to mood and anxiety disorders, heroin to neurological deficiencies, cocaine to immune system suppression, and alcohol to intimate partner abuse [12]. Additionally, female substance abusers, on average, have a lower level of education and lower rates of employment [13]. In light of these gender-specific concerns, physicians should give particular consideration to detecting substance abuse in women. Research in both humans and animals suggests that women may be more vulnerable to the reinforcing (rewarding) effects of stimulants, with estrogen possibly being one factor for this increased sensitivity [14].

Data suggest that females, compared to males, are more vulnerable to key phases of the addiction process that mark transitions in drug use such as initiation, drug bingeing, and relapse. Recent data also indicate that the female gonadal hormone estrogen may facilitate drug abuse in women. For example, phases of the menstrual cycle when estrogen levels are high are associated with enhanced positive subjective measures following cocaine and amphetamine administration in women [15]. Animal studies show that female rats are more sensitive to the rewarding, pain-relieving, and activity-altering effects of marijuana’s main active ingredient, delta-9-tetrahydrocannabinol (THC) [16]. Many of these differences have been attributed to the effects of sex hormones, although rodent research also points to the possibility that there are sex differences in the functioning of the endocannabinoid system, the system of brain signaling where THC and other cannabinoids exert their actions [17]. Table 1 highlights male–female differences from the perspectives of the most widespread substances of abuse.

## 4. Discussion

### 4.1. Addiction and Its Neurobiological Implications

The neurobiological underpinnings at the root of addiction development and progression are closely, albeit not solely, linked to specific brain areas such as the nucleus accumbens and the dorsal striatum. The former plays a meaningful role in the stimulus to undertake conduct from which the subject draws an initial sense of pleasure or reward; the dorsal striatum, on the other hand, comes into the picture in terms of escalating drug consumption, as well as conduct and behavioral patterns that are compulsive in nature [58,59]. While engaging in new pleasurable actions, such as taking a given substance for the first time, the endorphins in the nucleus accumbens determine the sense of reward or pleasure arising from such new experiences, whereas the sense of craving or the urge to seek such sensations again and again, i.e., the essence of addiction, are closely tied to the dopamine levels in both the nucleus accumbens and dorsal striatum [60]. Such patterns eventually result in dorsal striatum dopamine activation outpacing the response to the substance being taken in the nucleus accumbens, which in turns leads to lower levels of reward and pleasure even as the amount of such a substance grows [61]. Furthermore, the dorsal striatum gives rise to patterns of behavior that have the ability to unfold spontaneously, without individual control usually coming into being through the prefrontal cortex. Such automatic behavioral patterns are reportedly a cornerstone of addiction. Hence, as drug-taking transitions from occasional behavior to addiction, a different activation dynamic sets in within the user’s brain, based on dopamine activation in dorsolateral striatum rather than in the nucleus accumbens. Studies have shown how female rats and humans who transition from occasional drug consumers to addicts experience faster loss of conscious, controlled drug-intake than their male counterparts [62]. That is likely due to the fact that the dopamine release in the nucleus accumbens dwindles, which is the mechanism enabling the dorsal striatum to “take over” or supersede the subject’s behavioral patterns, causing them to become compulsive. Thus, the transition from occasional, controlled consumption to addiction is complete [63]. Such developments may be the reason why animal models show a tendency towards an initially weaker nucleus accumbens response to drug stimulation initially, as well as a relatively stronger and faster initial dorsal striatum response to substances, along with a weakening accumbens response following consumption of stimulants (cocaine in the studies available thus far) [64,65,66,67]. Higher levels of impulsivity and a greater likelihood to engage in addiction-like behaviors have been reported in male rats with lower levels of dopamine in the nucleus accumbens [68]. This may explain why female rats have been observed to engage in addiction-like behavior: lower cocaine-induced dopamine response in the nucleus accumbens. Both male and female rats that developed addiction-like behavior exhibited a reduction in cocaine-induced dopamine in dialysate in the nucleus accumbens [69]. As far as animal studies are concerned, it is worth noting the potential impact of the so-called “male bias”, which can be observed throughout pre-clinical research (i.e., the fact that male animals outnumber females by nearly six to one) [70], particularly in neuroscience [71]. The perceived higher level of hormonal variability or instability in female subjects has been cited to explain such a bias, but such a conclusion may be hasty and caused by stereotypical beliefs [72,73]. A recent broad-ranging meta-analysis [74] based on 293 articles and accounting for behavioral, morphological, physiological, and molecular traits in male mice and females seems to belie the “male bias” principles [74]; the study has in fact concluded that female mice showed no higher levels of hormonal variability or instability than their male counterparts. On the contrary, the opposite was found for several traits; significantly, both males and females showed 37% higher variability as a result of group housing. An even more recent sources relying on 311 articles and over 6000 data points [75] also ascribed no higher variability to female rats at any stage along the estrous cycle, and no sex differences were found to be linked to housing conditions on coefficient variations. Such sources agree that estrous cycle monitoring is not necessarily needed in neuroscience research, as does the most recent source, a late 2022 meta-analysis [76] relying on 263 studies and 4900 data points. Males were in fact found to exhibit a higher level of variability than unstaged females, for which learned fear is a major factor in that regard. Lower variability was in fact recorded in staged, not ovariectomized females. Individual housing led to a higher variability, and such effects were unaffected by sex. All in all, it is reasonable to conclude that variability in females is not affected by the estrous cycle, although it is female-specific modulator shaping and influencing behavior linked to fear and anxiety [76]. Therefore, studying neurological dynamics, key elements in substance addiction research, through a sort of “male lens” is in fact likely to detract from the level of reliability of addiction studies based on animal models. Just as important is the clarification of behavioral sensitization and its sex-induced patterns. Nonetheless, such neurological patterns are likely to play a pivotal role in sex-differentiated escalation of drug use ultimately climaxing into fully-fledged addiction. Supporting this notion, women who are smokers, for instance, exhibit a lower response in the ventral striatum than in the dorsal striatum to nicotine stimulation compared with male smokers [77,78]. Such reported neural correlates of sex- and gender- differences in substance use disorders, e.g., stress-related vulnerabilities affecting women more harshly, are highly significant. Furthermore, the various expressions of behavioral sensitization (i.e., the motor–stimulant response to a given substance following repeated drug use) [79] need to be researched further, if we are to devise ever more effective and consistently reliable therapeutic options. Such an aspect is indeed relevant, since sensitization is possibly among the behavioral contributors of craving [80]. Considering that females experience higher levels of sensitization to cocaine or amphetamine (even with lower doses) [81] than males, further research ought to dig deeper into sex-specific neurobiological implications of repeated cocaine administration. Studies focused on such multifaceted processes factoring in sex differentiation are still relatively few, although meaningful data have already been produced. Recent findings [82] centered around cocaine use reflect psychomotor stimulants eliciting behavioral responses only in female rats, but not males, along with estrogen-induced increases in dopamine activity in the striatum and nucleus accumbens. Interestingly, the level of sensitization registered throughout the drug exposure time span, and under conditions of enhanced estrogen, has been found to still linger when estrogen levels eventually subside [83]. Hence, the long-term effects of cocaine use with high estrogen levels are different, and clarifying such consequential mechanisms can greatly impact our knowledge of addiction and therapeutic capabilities. As for amphetamine use, faster metabolization has been reported in intact male rats, which means a smaller amount reaching the brain [84]. Still, when different doses of amphetamine are administered to males and females, resulting in the same brain concentrations, behavioral response in the latter is still more substantial [85,86]. The dearth of translational evidence, particularly due to the paucity of preclinical data on the role of ovarian hormones on the cocaine response and cocaine taking/seeking, should be addressed if we are to achieve more effective sex-targeted therapeutic pathways [87,88]. Additionally, the role of age in sex-specific drug abuse dynamics should not be discounted, and need further exploration. It is in fact noteworthy that such differences have been ascribed to both neurobiologic (meaning sex-related) and gender-related distinctions, that is, environmental external contributors closely linked to gender [89].

### 4.2. Beyond Neurobiology: A Wide Array of Gender-Related Contributors

Neurobiological data suggest that estrogen may facilitate drug taking by interacting with reward- and stress-related systems.

The administration of estrogen increases drug taking and facilitates the acquisition, escalation, and reinstatement of cocaine-seeking behavior in animal models. Despite this, it would be remiss to only account for biological factors when trying to delineate sex- and gender-based substance abuse dynamics; social and cultural backgrounds, i.e., gender elements, constitute majorly relevant influencing factors which affect men and women differently, also in terms of how individual responses to abuse substances and therapies turn out. Factors such as stigma, on-going interpersonal violence, substantial hurdles to accessing counseling and treatment, and inadequate social support for recovery weigh more heavily on female users [56]. The Institute of Medicine and the U.S. Preventive Services Task Force (USPSTF) recommends that clinicians screen and counsel for interpersonal violence [90]. It is essential to devise evidence-based prevention and screening guidelines to help clinicians identify those who need help and enable them to obtain the care they need [91]. Overall, women often report using substances to relieve stress or negative emotions. In addition, women are more vulnerable to developing substance use or other mental health disorders following divorce, loss of child custody, or the death of a partner or child. Some of the unique issues women who use drugs face affect their reproductive cycles. Some substances can increase the likelihood of infertility and early onset of menopause [91,92].

Substance use is also further complicated during pregnancy and breastfeeding [92,93]. Pregnant women using drugs, including tobacco and alcohol, can pass those drugs to their developing fetuses with harmful consequences. Similarly, new mothers using drugs can pass them on to their babies through breast milk and cause them harm. In pregnant women addicted to opioid pain relievers or heroin, the condition can result in neonatal abstinence syndrome (NAS) linked to opioid use. NAS occurs when heroin passes through the placenta to the fetus during pregnancy, causing the baby to become dependent on opioids. Symptoms include excessive crying, high-pitched cry, irritability, seizures, and gastrointestinal problems, among others [94]. The development of guidelines and gender-related drug abuse treatment must be shaped not only by biological differences but also by social and environmental factors, all of which can influence the motivations for drug use, the reasons for seeking treatment, the types of environments where treatment is obtained, the most effective treatments, and the consequences of not receiving treatment [95,96]. Guidelines outlining gender-specific approaches such as the ones issued by the European Monitoring Centre for Drugs and Drug Addiction (EMCDDA) [96,97] and the U.S. National Institute for Drug Abuse (NIDA) [2] are therefore needed to optimize the delivery of treatment. Estimates show that in the EU alone, as many as 30 million women and 50 million men in the 15–64 age range have experienced using an illegal substance in their lives. As mentioned earlier, the gender gap appears to be shrinking, especially among younger users [72]. While it would be premature to draw a set of distinctive traits as drug abuse among other gender identities, e.g., bisexual or transgender users, currently available findings reflect the difficulties such individuals face when seeking care. Despite the gradual shrinking of the gender gap, it is worth remarking that the difference between female and male users is still considerable when severe addiction issues are accounted for.

Females account for roughly 25% of problematic substance abusers; about 20% of those admitted to specialist drug treatment in Europe are female users [98]. Some data show how women may be more likely to seek care, probably due to pregnancy- or parenting-related needs. However, the record is still mixed, and women have been reported to be less inclined to pursue specialized treatment than their male counterparts due to a twofold type of stigmatization: drug use in general on the one hand, and being a woman with drug addiction issues on the other. Further research is urgently needed in order to clarify the degree to which this “treatment gap” plays a role in the recovery prospects of countless people with addiction issues, within different regions and sub-groups in Europe and all over the world [98].

### 4.3. Delineating More Effective Avenues of Care by Taking into Account Gender Implications

The fundamental differences between male and female users are noteworthy in terms of social make-up and distinctive traits, walk of life and socioeconomic background, and last but not least, the unfolding of drug use patterns [99]. All such elements determine the implications and consequences of substance abuse as well as the scope and progression to addiction. Providing adequate and more effective responses to such gender-specific issues can indeed make a difference by convincing prospective patients that getting treatment is the way to go, since all aspects of one’s individual and family life are likely to improve as a result. It is worth briefly elaborating on the distinctive sex- and gender-specific concerns, despite the fact that many addiction treatment services are still fundamentally male-oriented and thus may fall short in terms of providing sex- and gender-tailored therapeutic pathways [100].

Firstly, stigma hits female users harder than males, likely due to the fact that females engaging in drug abuse are still often perceived as disregarding or subverting consolidated gender roles, i.e., the expectations of societies on women as mothers and caregivers [100,101]. Thus, the stifling sense of stigma can be internalized and magnify feelings of guilt, self-loathing, and shame; against such a backdrop, the uneven, discriminatory nature of gender-unspecific treatment programs may disincentivize or even deter female users from trying to access care [102].

As mentioned earlier, female users report having experienced childhood trauma incidents (such as sexual abuse, physical assault, and the like) in greater numbers than men; intimate partner violence and other forms of gender-based violence in adult age have also been reported more frequently among women users. Drug use may in fact have been viewed as a means to cope with the trauma caused by sexual violence [103]. Gender-based violence within the setting of substance abuse is substantially more common among female users, e.g., through the coerced involvement in the sex trade or in the context of intimate relations. Instances of intimate partner violence are believed to be increased when one or both partners have substance use disorders [104]. In addition, drug-facilitated sexual assaults, i.e., the sexual victimization of anyone under the effects of drugs, whether these substances were consumed voluntarily or without the subject’s knowledge or consent, is more likely to involve female users, who are also more likely to trade sex for drugs or money [104,105].

Therefore, against such a backdrop, it stands to reason that mental health issues, e.g., post-traumatic stress disorders (PTSD), anxiety, and depression, are more frequently found among female users [106,107]. An underlying mental health issue prior to the development of substance abuse is also more frequently reported among women with psychiatric comorbidities. This means that female users with a dual diagnosis are more likely to be excluded from certain treatment services due to such a condition [107,108,109]. In fact, a major hurdle along the path to treating comorbid SUDs and psychiatric issues is the separation of mental health treatment services and addiction treatment facilities [109]. Hence, each treatment service is likely to lack proper resources and expertise for tackling both disorders concurrently and synergistically. That in turn may lead to various therapeutic approaches under different regulatory frameworks and inconsistent funding, to the detriment of effectiveness. Another factor worth mentioning is the COVID-19 pandemic and the deep changes it caused in drug trafficking and consumption dynamics [108,109]; socioeconomic stress contributors of considerable magnitude have added to the risk of mental health comorbidities in those with substance use disorders [106,107,108,109,110].

Social and economic factors often affect female users more severely, in light of the higher rates of unemployment and lower income levels overall compared to males. Substance abuse treatment services may come with a cost, if services are not totally covered by national health systems or insurance plans [110,111,112]. Less social support is often available for female users; data show that women with addiction issues are more likely to come from families with already entrenched issues of substance abuse. Women are also more likely to have partners with a history of substance abuse themselves [111,112]. Such a factor should not be taken lightly, in light of the fact that having a partner with addiction issues is significant and impactful in terms of drug use initiation, progression into addiction, and even relapse. It may also affect women’s risk of exposure to blood-borne viral infections and violence. Partners with addiction issues are less likely to provide support for their partners seeking care; moreover, the risk of damaging or losing their relationship if they were to seek help can deter female users from doing so. The presence of children can play a significant role as well; among addicts seeking treatment, women live with their children much more often than men [113]. Hence, childcare services, or lack thereof, can constitute a major hurdle for women when trying to seek care. It is therefore essential in order to guarantee and maintain the patient’s mother–child relationships, since that can greatly and decisively contribute to their recovery prospects [114].

## 5. Conclusions

Although research findings outlining possible sex differences in the metabolism of common substances of abuse are still rather inconclusive, evidence points out that ovarian hormones are modulators of plasma levels of cocaine, and are reported to be higher in women [115,116]. Certainly, a thorough analysis has to take into account the high level of inconsistency and heterogeneity between the sexes in terms of key metabolic processes. Moreover, multiple metabolic pathways for the clearance of substances, as contributing factors, can outweigh sex differences in drug metabolism. Gender-related peculiarities are also of utmost importance and must not be discounted; the set of multifaceted and highly complex, and frequently overlapping issues faced by many female drug users call for concerted, multidisciplinary measures and services based on coordinated and integrated measures. Gender-specific pathways and approaches are of utmost importance in order to provide comprehensive care and meet the unique needs of female substance users. Such needs can only be met by thorough service design and implementation: funding provision and subsidization for non-economically viable patients, structure and organization, location, adequate staffing, multidisciplinary care, and interventions.

More research is warranted to determine whether such programs should be female-only or mixed gender (but necessarily including specifically tailored services for women). Undoubtedly, community agencies such as child-care facilities and health care providers need to be able to rely on targeted specialized training designed to raise awareness for the purpose of identifying women who use drugs and put in place timely interventions or referrals (to psychiatric services, for instance), as necessary.

From a medicolegal perspective, it is of utmost importance to take into account the whole picture constituting each patient profile, which certainly includes sex-related factors and contributors, when outlining a therapeutic approach. Failure to execute this may undermine or deprive patients of their best prospects for recovery overall, and could therefore lead to negligence-based malpractice allegations, in light of the currently available scientific findings representing best practices with which clinicians need to comply when caring for patients with substance use disorders.

## Figures and Tables

**Table 1:** Noteworthy male–female differences from the perspectives of the most widespread substances of abuse.

Substance	Most Widely Reported Symptoms and Differences		Relevant Underlying Dynamics
	Male Users	Female Users	
Marijuana (Cannabis)	More intense substance-induced effects (“high”) have been observed, while adolescent male users reportedly experience poor family relationships and school issues more frequently than girls [18,19]	Spatial memory impairment has been reported in females more often than in their male counterparts. Possible higher risk of brain structural abnormalities arising from repeated/regular marijuana use. Animal models have shown how delta-9-tetrahydrocannabinol (THC, the chief psychoactive constituent of cannabis) can exert a greater effect on female users in terms of rewarding, pain-relieving, and activity-altering potential [20].	Sex hormones are reported to be determining factors of such differences; endocannabinoid system-related sex differences, i.e., within the system of brain signaling where THC and other cannabinoids exert their actions, have been reported as well [21,22,23].
Cocaine	Male users are more likely to report blood flow abnormalities in the brain’s frontal lobes [24,25]. Human studies accounting for cocaine users vs. a matched control group point to male users possibly tending more towards sensation seeking and behavioral risk-taking and displaying more impulsivity than females [26].	Studies involving both humans and animals point to female users being more susceptible to the rewarding and reinforcing action of cocaine and other stimulants, which may be due to estrogens [24,25]. Greater neural activation to cocaine cues has been reported in female users as well [27]. Females reportedly exhibit higher levels of behavioral sensitization than males [28,29], and may be more sensitive than men to cocaine’s effects on the heart and blood vessels.	In animal studies, females are more inclined to start taking cocaine—and take it in larger amounts—than males [22]. In contrast, female and male cocaine users show similar deficits in learning, concentration, and academic achievement, even if women had been using it longer [24].
Methamphetamine (METH)	Male users are more likely to switch to another drug when they lack access to methamphetamine [30,31]. In addition, male METH users have been found to have larger superior frontal cortices; such a finding seems to point to lower levels of dendritic pruning during adolescence [32]. More severe neuroinflammation in male recent users may be reflected by even larger volumes of frontal corticles: this may be associated with higher impulsivity levels. Lower cerebral glucose metabolism levels in the right superior frontal white matter, affecting executive function, has been reported abstinent male METH users [33]	Female METH users exhibit larger nucleus accumbens; this may point to estrogen mediating a protective glial response [32]. Unlike male METH users, females exhibit smaller and thinner frontal cortices, which may entail higher impulsivity levels, in turn suggesting greater neurotoxicity to these brain regions. Relatively larger nucleus accumbens suggest an estrogen-mediated neuroprotective glial response [32]. Women tend to begin using methamphetamine at an earlier age than do men and tend to develop more severe dependence [30]. Methamphetamine treatment reportedly has higher success rates in female users [30].	Male users exhibit larger volumes in the right superior frontal cortex than female counterparts, and this points to sex as a likely modulator of METH impact on brain morphometry. Motivations for use seem to vary between users of opposite sexes: female users are motivated to use METH for higher energy levels and to counter fatigue linked to work-related strain, home and child care, or family pressure [31,34]. Another major contributor is weight loss [31]. Higher rates of co-occurring depression have also been reported among female users. Male users turn to METH in an attempt to improve their sexual performances more often than women; being able to work more and more intensely is another major motivating factor for males, along with the urge to experiment new “highs” or replace previously used substances [35].
MDMA (Ecstasy)	Stronger hallucinatory effects have been reported in male users [36]. Male users are more sensitive to the acute physiological effects of ecstasy/MDMA [37]. Both men and women show similar increases in aggression a few days after they stop using MDMA [25,26]. Higher blood pressure increases have been reported in MDMA male users [38,39].	Female users are reportedly more sensitive than males to the acute and subacute physical and psychological effects of ecstasy/MDMA and long-term alterations in aspects of 5-HT functioning [40], whereas sensitization levels appear to be similar [41]. Higher rates of depression following MDMA last use have been reported in occasional female users [36,37] a few days after use, although females homozygous for the l-allele of the 5-HTTLPR (particularly polydrug ecstasy users) have been found to experience a mild reduction in self-rated depressive feelings [42].	MDMA can interfere with the body’s ability to eliminate water and decrease sodium levels in the blood, causing a person to drink large amounts of fluid [42]. In rare cases, this can lead to increased water in the spaces between cells, which may eventually produce swelling of the brain with possible lethal consequences [42,43]. Young women are more likely than men to die from this reaction, with almost all reported cases of death occurring in young females between the ages of 15 and 30 [42]. MDMA can also interfere with temperature regulation and cause acute hyperthermia, leading to neurotoxic effects and even death [37,38,39].
Heroin	Male users reportedly tend to use larger amounts of heroin and for longer, and are more likely to inject it than their female counterpart users [44,45].	One study indicates that women are more at risk than men for overdose death during the first few years of injecting heroin, although it is still unclear exactly why this might be the case [45,46]. Women who inject heroin may be more likely than their male counterparts to also use prescription drugs—a dangerous combination. Women who do not overdose within these first few years are more likely than men to survive in the long term. This could be due to differences in treatment and other environmental factors that impact heroin use [47,48].	Women have been reported to experience more cue-induced craving than male cocaine users. Females were more likely than males to experience mental health issues with the exception of antisocial personality disorder (ASPD) [49]. Females also had a higher prevalence of suicidal thoughts and behaviors than males [50]. Most women who inject heroin point to social pressure and sexual partner encouragement as factors. [45,46].
Ethanol	Men have higher rates of alcohol use overall, which includes binge drinking [51]. Girls ages 12 to 20, however, have reportedly slightly higher rates of alcohol misuse and binge drinking than males [52]. Striatal dopamine release—which is reflective of activation of the brain reward pathways—is higher in men relative to women in response to alcohol [53].	Similar amounts of alcohol result in higher blood ethanol concentrations in women who can therefore become intoxicated from smaller quantities, likely because of gastric tissue activity differences [54]. That can be specifically explained by the activity of alcohol dehydrogenase (i.e., the enzyme that metabolizes ethanol), which has been found to be lower in the gastric mucosa of female users [55].	Comparing people with alcohol use disorders, death rates up to 100 percent higher have been recorded in women, including suicides, fatal alcohol-related accidents, heart disease, stroke, and hepatic illness/failure [56]. Furthermore, several major health issues are linked almost uniquely to female users. Heavy drinking, for instance, entails higher likelihood of engaging in unprotected sex, with all related risks (e.g., unwanted pregnancy or disease), and a higher risk of being victimized sexually. Even small amounts such as one drink per day are reportedly linked to higher risk of breast cancer, especially in postmenopausal women or those with a family history of breast cancer [56,57].

## Data Availability

The data presented in this study are available upon request from the corresponding author.
